# Interventions to reduce burnout among clinical nurses: systematic review and meta-analysis

**DOI:** 10.1038/s41598-023-38169-8

**Published:** 2023-07-06

**Authors:** Miran Lee, Chiyoung Cha

**Affiliations:** 1 Department of Nursing, Kwangju Women’s University, Gwangju, South Korea; 2 College of Nursing, Ewha Research Institute of Nursing Science, System Health & Engineering Major in Graduate School, Ewha Womans University, #202 Helen Building, 52 Ewhayeodae-gil, Seodaemun-gu, Seoul, 03760 South Korea

**Keywords:** Health services, Occupational health, Health care

## Abstract

Sporadic evidence exists for burnout interventions in terms of types, dosage, duration, and assessment of burnout among clinical nurses. This study aimed to evaluate burnout interventions for clinical nurses. Seven English databases and two Korean databases were searched to retrieve intervention studies on burnout and its dimensions between 2011 and 2020.check Thirty articles were included in the systematic review, 24 of them for meta-analysis. Face-to-face mindfulness group intervention was the most common intervention approach. When burnout was measured as a single concept, interventions were found to alleviate burnout when measured by the ProQoL (n = 8, standardized mean difference [SMD] = − 0.654, confidence interval [CI] =  − 1.584, 0.277, p < 0.01, I^2^ = 94.8%) and the MBI (n = 5, SMD = − 0.707, CI = − 1.829, 0.414, p < 0.01, I^2^ = 87.5%). The meta-analysis of 11 articles that viewed burnout as three dimensions revealed that interventions could reduce emotional exhaustion (SMD = − 0.752, CI = − 1.044, − 0.460, p < 0.01, I^2^ = 68.3%) and depersonalization (SMD = − 0.822, CI = − 1.088, − 0.557, p < 0.01, I^2^ = 60.0%) but could not improve low personal accomplishment. Clinical nurses' burnout can be alleviated through interventions. Evidence supported reducing emotional exhaustion and depersonalization but did not support low personal accomplishment.

## Introduction

Burnout, first described by Freudenberger^1^, is a negative condition characterized by the gradual depletion of physical, emotional, and mental energy due to excessive work^2^. Maslach (1976) later conceptualized burnout as a multidimensional syndrome characterized by emotional exhaustion, depersonalization, and diminished personal commitment^3^. Burnout occurs during the maintenance of interpersonal relationships and is most prevalent in the fields of nursing, medicine, and education, which deal directly with many people^3^.

Nursing is an occupation that experiences one of the highest rates of burnout^4^. Nurse burnout is defined as a physical, psychological, emotional, and socially exhausted status caused by unsuccessfully managed job stress and limited social support^5^. The globally pooled prevalence of nurse burnout is 11.2%^6^. However, in other studies classifying burnout symptoms, nurse burnout was as high as 40.0%^7,8^. Moreover, nurse burnout in the post-COVID-19 pandemic era has worsened. In a recent study, nurse burnout was as high as 68.0%^9^.

The factors that contribute to burnout are diverse and intricate. Occupational stress is the most influential factor^10^. The causes of nurse burnout were excessive workload; lack of staffing; role conflict; low autonomy; time pressure; interpersonal conflict between patients, guardians, and medical staff; and absence of leadership support^11^. Burnout can have a significant impact on the group and the organization, so prevention and action are required^2^. The impact of nurse burnout is significant in that it not only negatively influences nurses but also patients and healthcare organizations^5^. Nurse burnout is associated with low-quality care, a threat to patient safety^12^, medication error^13^, and an extended patient hospital stay^14^. Nurses who experience burnout have physical symptoms, such as headache, fatigue, hypertension, and musculoskeletal problems^5^, and psychological symptoms, such as depression, sleep disorders, and difficulty concentrating^15^. Exhausted nurses may also experience behavioral disorders that negatively affect their health, such as smoking and drinking alcohol^5^. Nurse burnout might lead to the turnover^16^ and a subsequent burden to healthcare organizations^11^.

Nurse burnout has been a frequently investigated topic owing to its high prevalence and detrimental impact. However, systematic reviews and meta-analysis studies were focused on the description of the nurse burnout phenomenon such as the prevalence of nurse burnout^7^, burnout level and risk factors^17^, and burnout-related factors in nurses^18^. Previous systematic reviews or meta-analysis studies that evaluated the effects of burnout programs were limited to mindfulness training^19^ and coping strategies^20^. However, various programs, such as yoga, communication skills, stress management, mindfulness, meditation, and cognitive behavioral therapy, were implemented independently or in combination, and the level of evidence varied^21,22^. Nurse burnout interventions should be evaluated inclusively to understand their current effectiveness in reducing burnout among nurses. Previously conducted systematic reviews and meta-analyses on burnout interventions inclusively evaluated health professionals, which included nurses and medical doctors as participants^22,23^. However, nurses and medical doctors have different job descriptions^24^ and different patterns of burnout^25^. Accordingly, to retrieve evidence for nurse burnout programs, the analysis should be refined to interventions specifically designed and implemented for nurses.

Furthermore, burnout has been measured in many ways. Burnout could be measured as a single concept^26–28^, though it is often measured as three dimensions based on the International Classification of Disease-11 (ICD-11). The most frequently used measure is the Maslach Burnout Inventory (MBI), which lists three areas of burnout: emotional exhaustion, depersonalization, and low personal accomplishment^23,29^. Some studies used the total score of the MBI and others used the three areas of burnout with some variations^30,31^. To be inclusive, burnout interventions should be evaluated by including studies that used burnout as a single concept and as three dimensions. Per this understanding, we aimed to analyze burnout interventions for clinical nurses.

## Methods

### Design

This study is a systematic review and meta-analysis study on the effects of burnout reduction programs for clinical nurses. We followed the Preferred Reporting Items for Systematic Reviews and Meta-Analysis (PRISMA) guideline^32^.

### Eligibility criteria

We used the PICO-SD (Population, Interventions, Comparison, Outcome—Study Design) framework to organize our research question: What is the effect of an intervention on reducing burnout among clinical nurses? Detailed information regarding the eligibility criteria is described in Table 1. We selected articles published between 2011 and 2020 to yield results that reflected the reality of burnout intervention effects.

**Table 1:** Eligibility criteria.

	Inclusion criteria	Exclusion criteria
Population	Registered Nurses or Licensed Practice/Vocational Nurses providing direct care to their patients in hospitals	Studies with nurses who did not provide care independently or worked at outpatient clinics
Interventions	Any type of program that aimed to reduce nurse burnout	
Comparison	Inactive control group that did not receive an intervention or received usual care, or an active control group that received an alternative intervention for burnout	Studies without comparison groups
Outcome	Burnout	Studies that did not provide information on intervention results such as mean or standard deviation
Study Designs	Randomized controlled trial or quasi-experimental study	All other methodological studies
Language	English or Korean	Studies that did not provide original content

### Search strategies

Nine search engines were utilized: seven global search engines in English (PubMed, CINAHL, PsycINFO, Scopus, ProQuest Dissertations & Theses (PQDT) Global, EBSCO, and Cochrane Library) and two domestic search engines in Korean (RISS, KISS). The search terms were “nurse*” and “burnout” and a combination of (Nurses OR nurse* OR registered nurse* OR healthcare provider* OR nursing staff OR healthcare worker* OR health care provider* OR health care worker* OR health personnel* OR health professional*) AND (burnout OR burn-out OR burn out) AND (treatment* OR intervention* OR program* OR therapy OR training OR exercise* OR practice* OR mindfulness OR meditation OR massage OR yoga).

### Study selection and data extraction

Endnote 20.0 was used to manage retrieved studies and screen the redundant ones. After retrieval of the studies, titles and abstracts were reviewed to remove irrelevant studies. A full-text review of the studies was conducted afterward. Throughout the process, we worked independently and met weekly to discuss the process and select the studies.

### Risk-of-bias assessment

To evaluate the risk of bias, we used the Cochrane’s Risk of Bias 2.0 (RoB 2.0) for the randomized controlled trials and Risk of Bias in Non-randomized Studies of Interventions (RoBINS-I) for the quasi-experimental studies. Discrepancies were resolved through discussion. In addition, a funnel plot was utilized to evaluate the possibility of publication bias.

### Data synthesis and meta-analysis

For the systematic review, tables were used to classify article contents for descriptive analyses. For the meta-analysis, the R-4.1.1 program for Windows was used. In 16 articles, burnout was measured as a single concept using various instruments, while in 11 articles, burnout was measured as three dimensions: emotional exhaustion, depersonalization, and low personal accomplishment. Meta-analysis was conducted with the fixed effect model and the random effect model with 95% confidence interval, pooled mean differences, and weight of each article for each meta-analysis. The heterogeneity of the articles was calculated using the I^2^ index. This research was exempted after review by the institutional review board at the institution of the principal investigator.

## Results

### Study selection

**Figure 1:**
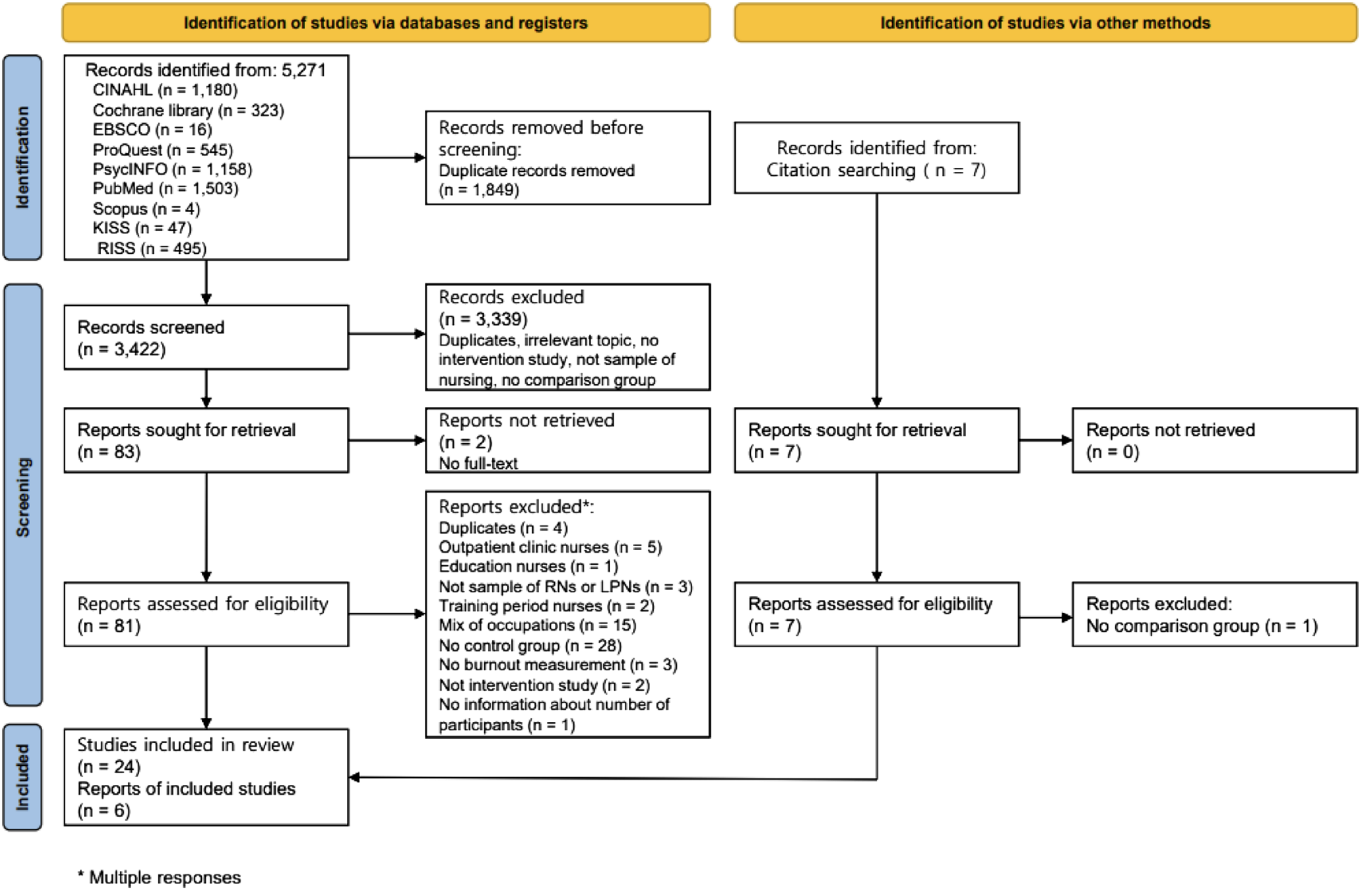
Study selection.

We retrieved 5271 articles from the initial search. After reviewing the title and abstract, 5188 were excluded (duplicates, no intervention study, no comparison group, not target population). During the full-text review, 59 articles were excluded (no full-text, duplicates, no intervention study, no comparison group, not target population). Through reference check, six articles were included. Finally, 30 articles were included in our final analysis (Fig. 1).

### Study characteristics

The characteristics of studies and interventions are described in Table 2. Of the 30 articles, 12 were randomized controlled trials^26,28,33–42^ and 18 were quasi-experimental studies^27,30,31,43–57^. Nineteen studies were conducted in Asia (Korea = 14, China = 3, India = 1, and Japan = 1). The types of publication were journals (n = 26) and thesis (n = 4). Participants were mostly women, with the female gender ranging from 71.9 to 100%. The age range of the participants was 24–46 years. There were between 21 and 296 participants, for a total of 1935, with 975 in the experimental group and 960 in the control group.

**Table 2:** Characteristics of the included studies.

Author, year (Country)	Design	Risk of bias	Participants (Number, female ratio)	Mean age of participants	Measures	Intervention (Type, duration, mode, comparison)	Follow-up (f/u) time points
Ahn (2017)^37^ (Korea)	Quasi-experimental	Moderate	E = 15, C = 15 100%	N/A	MBI	Type: mindfulness-based stress reduction program Duration: 5 weeks Mode: face to face, group Comparison: waitlist	Pre, post
Alenezi et al. (2019)^38^ (Saudi)	Quasi-experimental	Moderate	E = 154, C = 142 N/A	N/A	MBI	Type: burnout prevention workshop Duration: 2 days Mode: face to face, group Comparison: no information about intervention	Pre, f/u (1, 3, 6 months)
Alexander et al. (2015)^27^ (USA)	RCT	Some concerns	E = 20, C = 20 97.5%	46.4	MBI	Type: yoga Duration: 8 weeks Mode: face to face, group Comparison: usual care	Pre, post
Bae et al. (2019)^39^ (Korea)	Quasi-experimental	Moderate	E = 17, C = 17 91.2%	31.7	MBI	Type: mindfulness-based stress reduction program Duration: 4 weeks Mode: face to face, group Comparison: waitlist	Pre, post, f/u (4 weeks)
Bagheri et al. (2019)^25^ (Iran)	Quasi-experimental	No Information	E = 30, C = 30 88.1%	33.2	MBI	Type: stress-coping & cognitive behavioral therapy Duration: 10 weeks Mode: face to face, group Comparison: no information about intervention	Pre, post, f/u (1 month)
Berger et al. (2011)^28^ (Israel)	RCT	Some concerns	E = 42, C = 38 100%	E = 49.3 C = 47.7	ProQoL	Type: reducing secondary traumatization Duration: 12 weeks Mode: face to face, group Comparison: waitlist	Pre, post
Choi et al. (2016)^40^ (Korea)	Quasi-experimental	Moderate	E = 34, C = 15 N/A	E = 24.0 C = 25.4	Pines, Aronson, Kafry (1981)	Type: empowerment program Duration: 2 days Mode: face to face, group Comparison: waitlist	Pre, post
Dincer et al. (2021)^21^ (Turkey)	RCT	Some concerns	E = 35, 91.4% C = 37, 86.5%	E = 33.5 C = 33.4	Pines & Aronson (1988)	Type: emotional freedom techniques Duration: 20 min Mode: face to face, group Comparison: waitlist	Pre, post
Duarte et al. (2016)^41^ (Portugal)	Quasi-experimental	High	E = 29, 89.6% C = 19, 84.2%	E = 38.9 C = 42.1	ProQoL	Type: mindfulness-based stress reduction program Duration: 6 weeks Mode: face to face, group Comparison: waitlist	Pre, post
Felker. (2013)^42^ (USA)	Quasi-experimental	Moderate	E = 17, C = 17 94.1%	40.3	MBI	Type: yoga Duration: 6 weeks Mode: face to face, group Comparison: waitlist	Pre, post
Jang (2019)^43^ (Korea)	Quasi-experimental	Moderate	E = 24, 75.0% C = 24, 87.5%	E = 26.1 C = 25.8	MBI	Type: workplace mutual respect program Duration: 4 months Mode: face to face, group Comparison: no information about intervention	Pre, post
Jang et al. (2015)^26^ (Korea)	Quasi-experimental	Moderate	E = 14, C = 15 100%	N/A	MBI	Type: group art therapy Duration: 8 weeks Mode: face to face, group Comparison: no information about intervention	Pre, post, f/u (4 weeks)
Kang et al. (2017)^44^ (Korea)	Quasi-experimental	Moderate	E = 15, C = 23 N/A	E = 27.9 C = 26.6	ProQoL	Type: self-reflection program Duration: 6 weeks Mode: face to face, group Comparison: no information about intervention	Pre, post
Kharatzadeh et al. (2020)^29^ (Iran)	RCT	Some concerns	E = 26, 92.3% C = 27, 88.8%	E = 41.0 C = 39.2	ProQoL	Type: emotional regulation training Duration: six 2-h sessions Mode: N/A Comparison: waitlist	Pre, post
Kil et al. (2016)^30^ (Korea)	RCT	Some concerns	E = 26, C = 30 100%	N/A	MBI	Type: self-cosmetology training program Duration: 3 weeks Mode: face to face, group Comparison: no information about intervention	Pre, post
Kim et al. (2016)^45^ (Korea)	Quasi-experimental	Moderate	E = 14, C = 18 71.9%	26.9	ProQoL	Type: overcoming compassion fatigue program Duration: 5 weeks Mode: face to face, group Comparison: waitlist	Pre, post
Kim et al. (2018)^22^ (Korea)	Quasi-experimental	Moderate	E = 23, 100% C = 24, 91.7%	N/A	OLBI	Type: group rational emotive behavior therapy program with group counseling Duration: 8 weeks Mode: face to face, group Comparison: no information about intervention	Pre, post, f/u (4 weeks)
Kubota et al. (2016)^31^ (Japan)	RCT	Some concerns	E = 50, 96.0% C = 46, 96.0%	E = 38.9 C = 40.0	MBI	Type: psycho-oncology training program Duration: 16 h (2 days) Mode: face to face, group Comparison: waitlist	Pre, f/u (3 months)
Lee et al. (2017)^46^ (Korea)	Quasi-experimental	Moderate	E = 18, C = 18 N/A	30.6	MBI	Type: violence coping program Duration: 4 weeks Mode: face to face, group Comparison: waitlist	Pre, post, f/u (4 weeks)
Luo et al. (2019)^47^ (China)	Quasi-experimental	High	E = 41, C = 46 97.7%	28.1	MBI	Type: record 3 good things Duration: 4 weeks Mode: mobile application Comparison: no information about intervention	Pre, post
Özbaş et al. (2016)^32^ (Turkey)	RCT	Some concerns	E = 38, C = 44 N/A	N/A	MBI	Type: psychodrama-based psychological empowerment program Duration: 10 weeks Mode: face to face, group Comparison: waitlist	Pre, f/u (1 month after intervention), f/u (3 month after intervention)
Rajeswari et al. (2020)^23^ (India)	RCT	Some concerns	E = 60, C = 60 80.0%	N/A	ProQoL	Type: accelerated recovery program Duration: 5 weeks Mode: N/A Comparison: routine activity	Pre, post, f/u (3, 6, 9, 12 months)
Redhead et al. (2011)^33^ (England)	RCT	Some concerns	E = 12, 83.0% C = 9, 78.0%	E = 39.4 C = 42.6	MBI	Type: psychosocial intervention training Duration: 8 months Mode: face to face, group Comparison: waitlist	Pre, post
Rhee et al. (2012)^48^ (Korea)	Quasi-experimental	Moderate	E = 13, C = 15 100%	E = 43.8 C = 41.5	MBI	Type: mindfulness-based stress reduction program Duration: 4 weeks Mode: face to face, group Comparison: no information about intervention	Pre, post
Sabancıogullari et al. (2015)^49^ (Turkey)	Quasi-experimental	Moderate	E = 33, 97.2% C = 30, 93.3%	E = 27.5 C = 29.6	MBI	Type: professional identity awareness development education program Duration: 10 weeks Mode: face to face, group Comparison: no information about intervention	Pre, post, f/u (6 months)
Shin et al. (2020)^34^ (Korea)	RCT	Some concerns	E = 25, C = 25 100%	E = 26.4 C = 26.6	ProQoL	Type: Patchouli oil inhalation Duration: 24 h Mode: N/A Comparison: pure sweet almond oil inhalation	Pre, post
Wei et al. (2017)^35^ (China)	RCT	Some concerns	E = 51, C = 51 86.0%	N/A	MBI	Type: active intervention and regular management Duration: 6 months Mode: N/A Comparison: regular management	Pre, post
Xie et al. (2020)^36^ (China)	RCT	Some concerns	E = 53, C = 53 100.0%	27.7	MBI	Type: mindfulness Duration: 8 weeks Mode: face to face, group Comparison: education	Pre, post, f/u (1, 3 months)
Yoo (2017)^50^ (Korea)	Quasi-experimental	Moderate	E = 21, 90.5% C = 27, 96.3%	E = 26.1 C = 26.5	ProQoL	Type: expressive writing program Duration: 5 weeks Mode: non-face-to-face, individual Comparison: no information about intervention	Pre, post
Yoon (2013)^51^ (Korea)	Quasi-experimental	Moderate	E = 25, C = 25 N/A	N/A	MBI	Type: happy arts therapy Duration: 4 weeks Mode: face to face, group Comparison: no information about intervention	Pre, post

*C* comparison group, *E* experimental group, *MBI* Maslach Burnout Inventory scale, *OLBI* Oldenburg Burnout Inventory, *ProQoL* Professional Quality of Life Scale, *RCT* Randomized controlled trial, *N/A* not available.

The most common interventions provided for burnout reduction were mindfulness-based stress reduction programs (n = 5) and face-to-face group format (n = 24). The duration of the intervention varied from one day to eight months. In most studies, control groups involved the waitlist group (n = 12) rather than an active control group. MBI (n = 19), ProQoL (n = 8) and others (n = 3) were the instruments used to measure burnout. Burnout was most often measured twice, before the intervention and immediately post-intervention. In three studies^37,38,44^, burnout was measured at baseline and follow-up only, not immediately post-intervention.

### Risk-of-bias

Risk-of-bias is described in Table 2. In general, the level of risk of bias for 12 randomized controlled trials was “some concern.” The level of risk of bias for the 18 quasi-experimental studies was “low risk of bias” for 15 studies, “moderate risk of bias” for two studies, and non-assessable due to limited information for one study.

**Figure 2:**
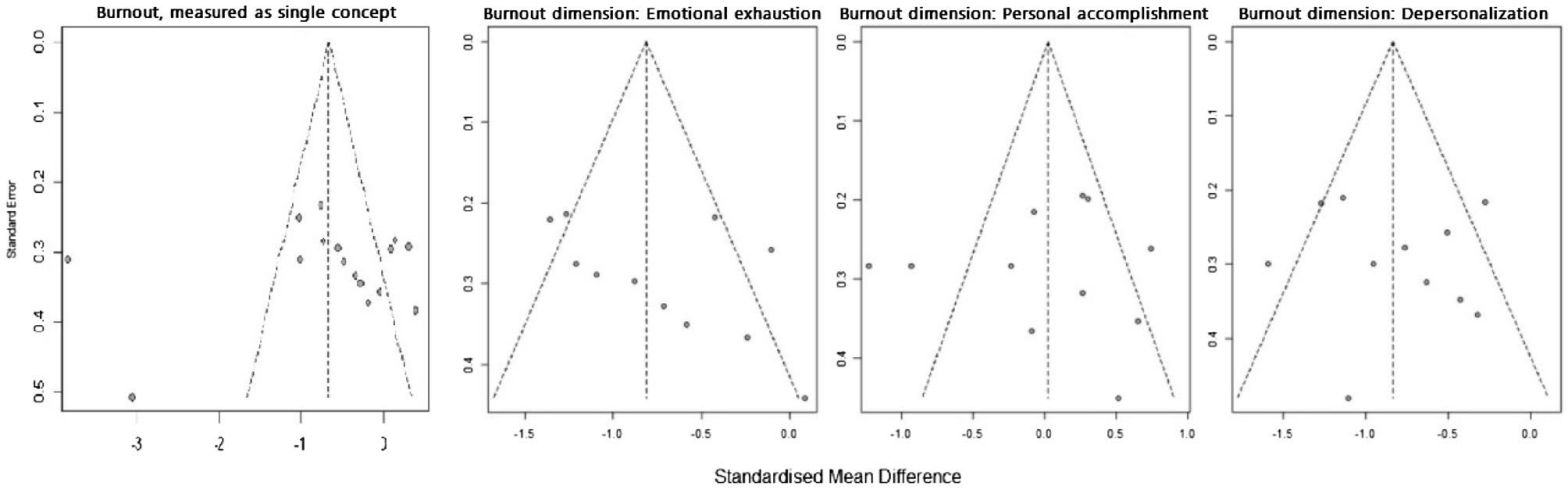
Funnel plots.

The risk of publication bias was evaluated using a funnel plot (Fig. 2). The plot is symmetrical when publication bias is at minimum^58^. Studies with a small sample size were on the lower side, while those with a large sample size were on the opposite side. The small number of articles used in our study was a risk factor because it could affect the precision of the results. Among 30 articles, three articles^37,38,44^ that did not conduct a post-test were excluded for meta-analysis. Sixteen articles measured burnout as a single concept^26–28,31,34,35,40,45–47,49–52,54,56^ and 11 measured burnout as three dimensions: emotional exhaustion, depersonalization, and low personal accomplishment^30,33,36,39,41–43,48,53,55,57^. There was one outlier among articles that measured burnout as a single concept.

### Meta-analysis

**Figure 3:**
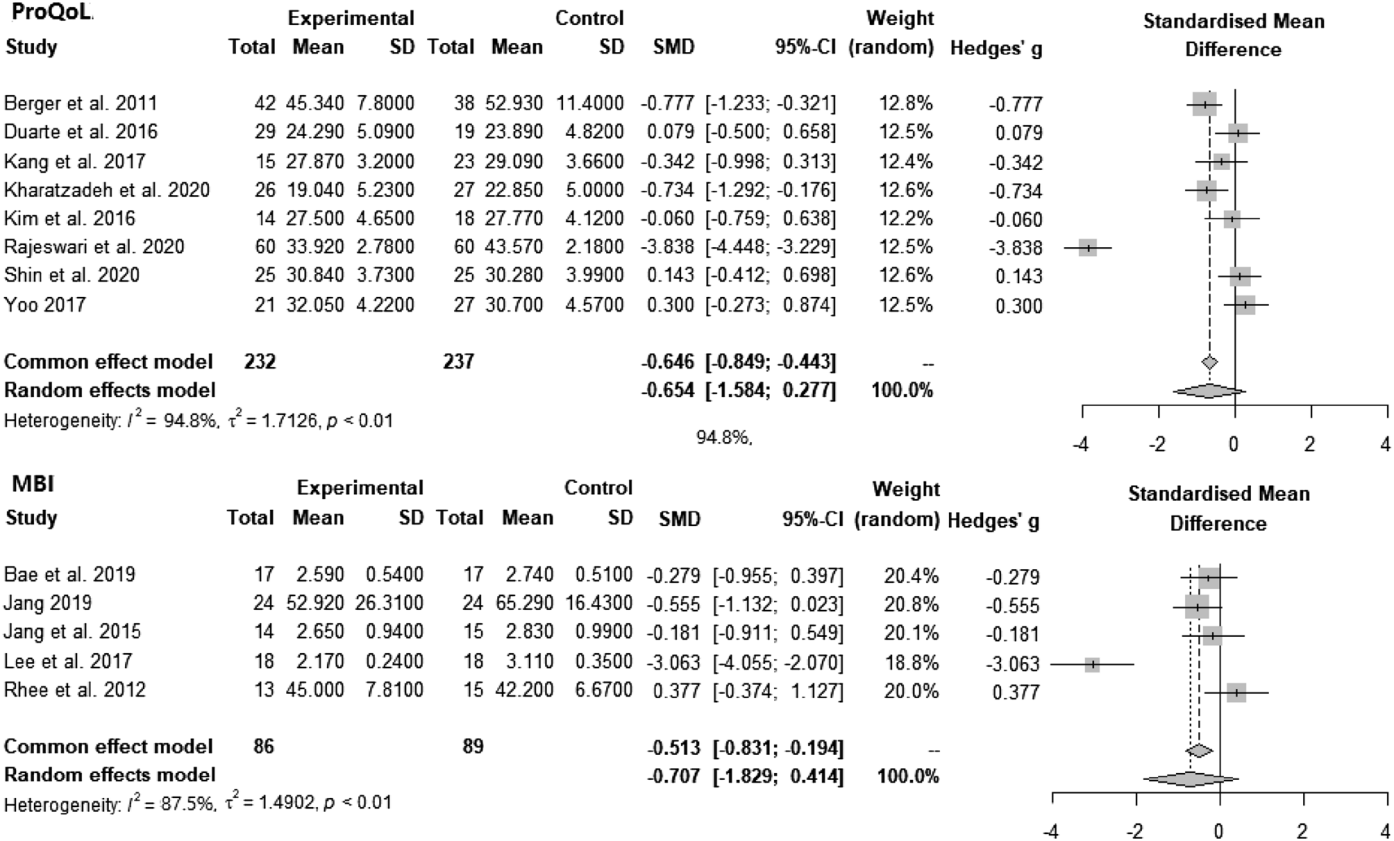
Forest plots: Effect of interventions on burnout measured by ProQoL and MBI.

Instruments that measured burnout as a single concept were ProQoL (n = 8), MBI (n = 5), burnout questionnaire (n = 2), and OLBI (n = 1). Meta-analysis of articles that used ProQoL and MBI are described in Fig. 3. For the articles that used ProQoL, the pooled analysis showed that intervention could statistically alleviate burnout (SMD = − 0.654, CI = − 1.584, 0.277, p < 0.01, I^2^ = 94.8%). For the articles that used the MBI, the pooled analysis showed that intervention could statistically alleviate burnout (SMD = − 0.707, CI = − 1.829, 0.414, p < 0.01, I^2^ = 87.5%).

**Figure 4:**
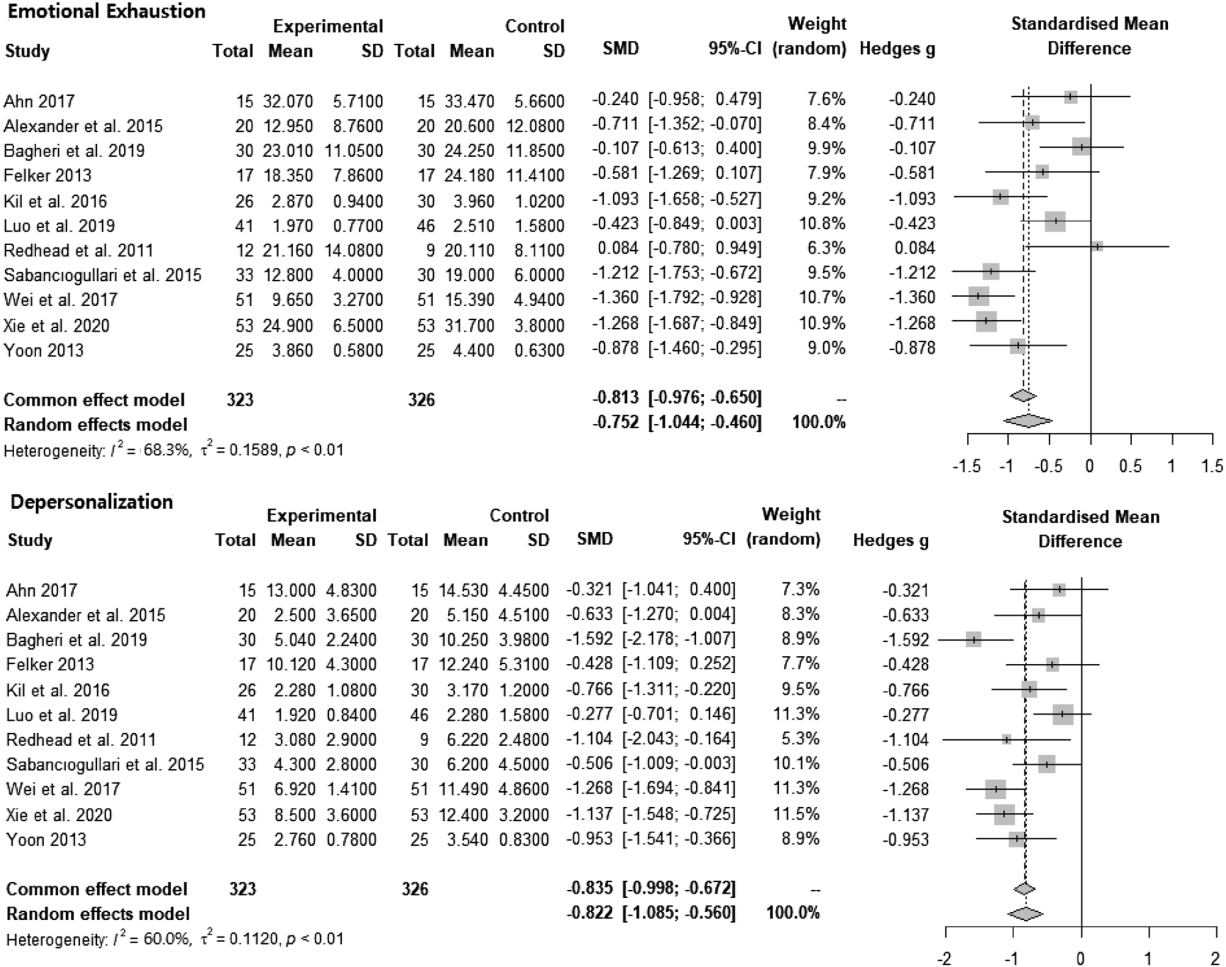
Forest plots: effect of intervention on emotional exhaustion and depersonalization.

The meta-analysis of burnout interventions as three dimensions (n = 11) is described in Fig. 4. The pooled analysis showed that interventions could statistically significantly reduce emotional exhaustion (SMD = − 0.752, CI = − 1.044, − 0.460, p < 0.01, I^2^ = 68.3%) and depersonalization (SMD = − 0.822, CI = − 1.085, − 0.560, p < 0.01, I^2^ = 60.0%). For improving low personal accomplishment, the pooled analysis result was not statistically significant.

## Discussion

In this systematic review and meta-analysis, we analyzed 30 and 24 articles, respectively. Among 30 articles, more than half (n = 19) were published in Asia. Although nurse burnout is a global phenomenon, the prevalence of nurse burnout studies conducted in Asia might indicate the significance of the issue of nurse burnout in Asian countries. This notion is supported by a recent meta-analysis study on the global prevalence of nurse burnout, which reported that Southeast Asia and the Pacific region had a significantly higher prevalence of nurse burnout among si× global regions^6^. In Asia, nurses encounter poor working conditions such as low nurse patient ratios^59^ and a rapidly aging population. High prevalence of nurse burnout in Asian countries might have drawn the nurse administrators and nursing scholars to research on nurse burnout interventions.

Our systematic review revealed that a mindfulness-based program was the most frequently used intervention for nurse burnout. Meta-analysis studies^19^ have shown that mindfulness-based programs are effective in reducing nurse burnout. However, burnout refers to a state of physical, mental, and social exhaustion that may require various interventions. A systematic review of health professional burnout programs revealed that a vast array of interventions have been adopted alone or in combination^24^. Although mindfulness-based programs are helpful in lowering burnout level, their role might be limited to managing burnout rather than preventing or managing situations for burnout^60^. In many cases, the causes of burnout are multifaceted, which include but are not limited to issues with limited manpower, working longer shifts, not having schedule flexibility, and responding to high work and psychological demands^11^. Systematic support to improve work environments and tailored programs to train nurses to prevent repeated situations are needed.

All articles were appraised for risk of bias. The most concerning realm for risk of bias in both the randomized controlled trials and quasi-experimental studies was bias in the measurement of outcomes that were appraised as “some concern” or “moderate risk of bias.” As burnout is a subjective concept, all the interventions used a self-reported survey to measure the outcome, leading to a moderate risk of bias. To overcome this, biological indicators for burnout could be utilized. However, we would like to note that people are experts in their own feelings and psychological health. In measuring psychological concepts such as burnout, the concept of risk of bias should be re-assessed.

In our meta-analysis of articles that measured burnout as a single concept with ProQoL and MBI, the results favored intervention. Similarly, results of previous meta-analyses of various burnout interventions provided to health professionals reported that burnout could be reduced^23^. In this study, the authors argued that various factors, such as coping strategies, emotional regulation skills, and resilience, were enhanced through diverse burnout interventions and bridged health professionals’ burnout to wellness. Likewise, various programs could be utilized solitarily or in combination to reduce nurse burnout.

When burnout was measured as three dimensions, emotional exhaustion and depersonalization were lowered, leaving no evidence for increasing low personal accomplishment. In contrast, a recent meta-analysis study on burnout intervention for primary healthcare professionals reported that interventions had beneficial effects on all three dimensions of burnout, including low personal accomplishment^61^. In the previous meta-analysis study, 78.5% of the participants were physicians, while only 20.1% were nurses. This was one of the most significant differences between the studies. The nature of the profession in achieving personal accomplishment may explain the differences in intervention effect on low personal accomplishment. Personal accomplishment for nurses may be more closely tied to a workplace system. For instance, a study that measured personal accomplishment found that it was positively correlated with aspects of the workplace such as control, community, fairness, and values^62^. In accordance with this argument, a meta-analysis that examined the long-term effect of burnout intervention on nurses found that improvement in low personal accomplishment lasted only six months, whereas improvement in emotional exhaustion and depersonalization lasted a year^20^. The authors of this study also explained that low personal accomplishment is difficult to change in the long term because it is reliant on the work environment. Another possible reason for the burnout intervention not favoring low personal accomplishment might be owing to the contents of the intervention focusing on problem-solving skills, such as stress reduction, coping with the problem, and empowering the participants, which are helpful for emotional exhaustion and depersonalization.

Implications for future research are suggested as follows. This study revealed that the majority of burnout interventions for clinical nurses were delivered as face-to-face group programs, which could be challenging to implement during a pandemic such as COVID-19. Combining online and offline burnout programs may be an option for reducing the risk of infection. Despite the fact that clinical nurses benefit from burnout programs, they may require consistent support and feedback to continue the program^63^. Continual active feedback may be necessary for the implementation and maintenance of the burnout program for clinical nurses. A number of scholars view burnout as three dimensions in line with the ICD-11 definition of burnout and meta-analysis studies on the prevalence and risk factors for burnout explained burnout as three dimensions^6,64^, meaning there is ample evidence on the dimensions of burnout. However, when examining the effect of burnout interventions, burnout is often measured as a single concept. Burnout interventions should be designed to target all three areas. Additionally, more time and effort might be needed to promote personal accomplishment.

## Limitations

In this study, we focused on nurses providing direct care in hospitals, excluding those who worked in outpatient clinics. Thus, our findings are limited to clinical nurses. The articles’ language was limited to English and Korean, half of which were in Korean. In addition, we limited our search to the past 10 years to reflect the reality of the burnout intervention effect, which may have caused selection bias. When the risk of bias was appraised, we identified some concerns, including moderate concerns. In addition, articles analyzed in this study used different instruments to measure burnout. We acknowledge the heterogeneity of the data, which is assumed by meta-analysis study. Thus, readers of this article should be aware of the risk of bias in the results and heterogeneity of the articles in instruments. The protocol of this systematic review and meta-analysis was not registered.

## Conclusions

Thirty articles were included in the systematic review and 24 in the meta-analysis. Most of the evidence for nurse burnout was based on face-to-face group programs, which could be transformed into a virtual space in the post-COVID-19 era. Pooled analysis suggested that interventions could reduce burnout when measured as a single concept and reduce the emotional exhaustion and depersonalization dimensions of burnout. However, we could not find evidence for burnout interventions effectively promoting personal accomplishment.

## Data Availability

The datasets generated and/or analyzed during the current study are not publicly available due to the IRB restriction but are available from the corresponding author on reasonable request.

## Abbreviations

CI: Confidence interval
ICD-11: International Classification of Disease-11
MBI: Maslach Burnout Inventory
N/A: Not available
OLBI: Oldenburg Burnout Inventory
ProQoL: Professional Quality of Life Scale
RCT: Randomized controlled trial
SMD: Standardized mean difference
